# Investigating the behavioural responses of sheep used for teaching veterinary undergraduates

**DOI:** 10.1002/vetr.5669

**Published:** 2025-09-30

**Authors:** Sander Prins, Kathryn Ellis, Jayne Orr, Dorothy McKeegan

**Affiliations:** ^1^ School of Biodiversity One Health and Veterinary Medicine University of Glasgow Glasgow UK; ^2^ UMR Herbivores VetAgro Sup, Université Clermont Auvergne Saint‐Genès‐Champanelle France

**Keywords:** animal welfare, habituation, sheep, stress behaviour, veterinary education

## Abstract

**Background:**

The use of animals for teaching veterinary medicine to veterinary students could induce stress and therefore affect the welfare of these animals.

**Methods:**

Six Easycare ewes were used for clinical examination classes for veterinary students once a week, for 5 consecutive weeks. Sheep behaviour was video recorded before, during and after these classes, and subsequently categorised into maintenance or stress behaviour. Data were analysed to describe behaviour observed before, during and after teaching sessions, to investigate if habituation to stress developed over the 5 weeks and to explore what factors (day, teaching session within the day, number of people in the pen) influenced stress behaviour.

**Results:**

Individual differences in both the number of stress behaviours exhibited and the overall stress response were observed between sheep. Overall, the total number of stress behaviours observed decreased between Day 1 and Day 5. Behaviour seen after teaching varied between days, with sheep spending more time lying on Days 2 and 3 and more time eating on Days 4 and 5. Sheep spent less time standing on all days after being used for teaching.

**Limitations:**

The individual differences between sheep, in combination with the low number of sheep and the short observational time, may have affected the outcomes of the study.

**Conclusion:**

Sheep experience stress when used in clinical examination classes, with variation between individuals. The lower number of stress behaviours observed in the last 2 days may suggest habituation to these classes. The increase in lying behaviour after teaching may indicate potential tiredness.

## INTRODUCTION

The ability to perform a clinical examination is a Day 1 competency, as described by the Royal College of Veterinary Surgeons and the European Coordinating Committee on Veterinary Training.^1^, ^2^ In the undergraduate veterinary curriculum at the University of Glasgow, all students are trained to carry out clinical examinations on sheep, as well as cattle, horses and companion animals. During clinical examination training, sheep are used in accordance with the three R's (replacement, reduction, refinement), as first proposed by Russel and Burch in 1959 (and subsequently refined),^3^ and guidelines such as those provided by the American Association of Veterinary Medical Colleges.^4^ Although the sheep used for teaching are handled with respect and care during these classes, being restrained for handling and contact with the students could cause a stress response in the sheep due to their fear of novelty and people.^5^, ^6^

The behavioural response of a sheep to a stressor is influenced by physiological pathways, such as activation of the endocrine and nervous pathways, which can vary between individual sheep.^7^ The behavioural responses to stress demonstrated in sheep are partly because they are prey animals.^6^, ^8^ The experience of fear (e.g., fear of people) can lead to behavioural responses such as escape or immobilisation.^5^, ^9^, ^10^ The manifestation of the behavioural response can also be partly influenced by the breed, sex and reproductive status of the sheep.^11^, ^12^, ^13^, ^14^ In addition to specific stress behaviours, a reduction in normal maintenance behaviours (such as rumination, lying, social behaviour, eating/drinking) can also be observed in response to a stressor.^15^ Habituation or aversion to a particular stressor has been reported in sheep and may depend on the conditions under which the animals were reared and on previous human–animal interactions.^16^, ^17^, ^18^, ^19^, ^20^, ^21^

The stress response of sheep used for training veterinary students in clinical skills, such as clinical examination, and any potential habituation has not been described before, and there is limited work in other domestic species. In cattle, stress responses have been described to be lower when simulator‐trained students performed rectal palpation, compared to the non‐simulator‐trained students.^22^ The objective of this study was, therefore, to address the knowledge gap regarding the behavioural responses of sheep used in clinical examination teaching. The main aims were (i) to determine the levels of stress and signs of habituation experienced by sheep during teaching; (ii) to investigate the differences in maintenance behaviour before, during and after teaching; and (iii) to investigate the factors influencing stress behaviour. It was hypothesised that sheep would show stress behaviour during teaching and that habituation would occur after being used for teaching once a week for 5 weeks. It was also hypothesised that there would be a difference in maintenance behaviour in the period after being used for teaching compared to the period before teaching and that factors other than the use for teaching would influence stress behaviour.

## MATERIALS AND METHODS

Six non‐pregnant, 6‐year‐old ewes (Easycare, mean weight 67 kg [range 50–83.5 kg]) were used in the clinical examination classes. The ewes were acquired from the commercial flock of the University of Glasgow farm and had not previously been used in veterinary teaching classes or for research. They arrived 1 week before the start of the study and were housed within the on‐campus teaching hospital in two group housing pens (6 × 2.7 m, each pen contained three ewes) to acclimatise. The pens were bedded with straw, the ewes had ad libitum access to hay (two group troughs were available per pen) and water, and they were fed 0.2 kg of concentrates per head each morning. The ewes in each pen were in visual contact with the other pen. Apart from an initial clinical examination by the university clinician on arrival, no further handling or examination was performed before the experimental period. The study was approved by the Animal Welfare and Ethics Committee of the School of Veterinary Medicine at the University of Glasgow (EA 52/21).

### Clinical examination classes and students

**FIGURE 1:**
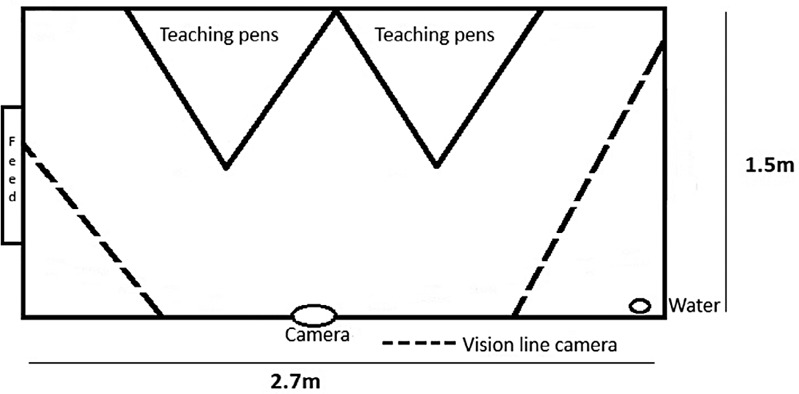
Design of the housing pen when small triangular pens are set up for the teaching session. The vision lines of the camera are also shown

The five clinical examination classes were held once a week, on the same day and same time of day each week, for 5 consecutive weeks from mid‐January to the end of February 2022. Each clinical examination class was divided into two 1‐hour parts. During each hour, only three sheep were used (one sheep from one housing pen, two sheep from the other housing pen), so that all six sheep were used over 2 hours. For the clinical examination classes, two small temporary triangular pens (1.8 m on each side), constructed of hurdles, were placed within each housing pen. Approximately 10–15 minutes before the start of the class, three sheep were placed in three of the four small triangular pens (one sheep per triangular pen) (Figure 1). The other three sheep remained loose in the housing pens (one from one pen and two from the other). After 1 hour of clinical examination, the sheep in the triangular pens were replaced by those that had not yet been used.

**FIGURE 2:**
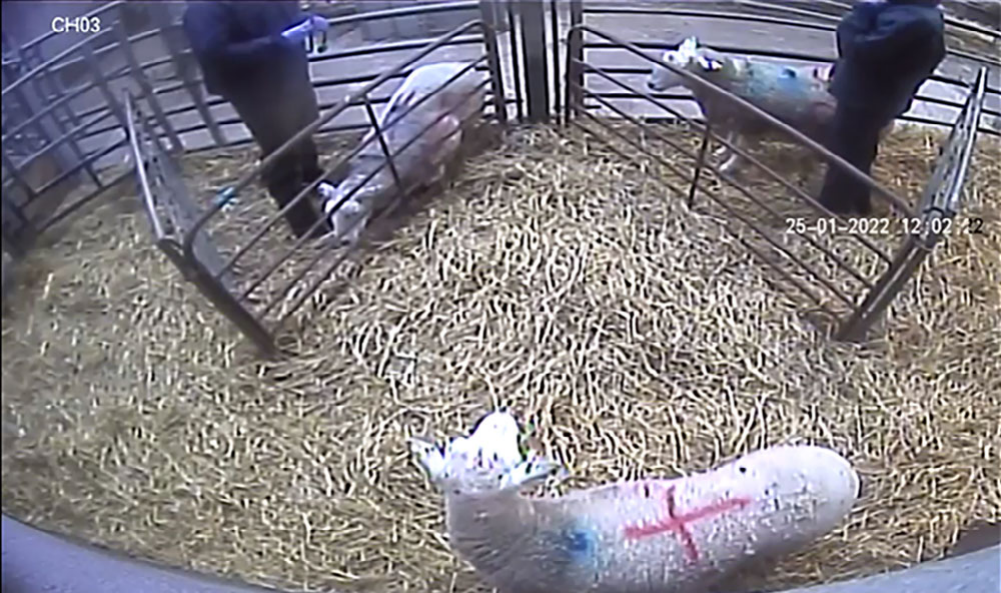
Example of the camera perspective when the sheep were used for teaching

Eight students were present during each teaching hour and had the opportunity to perform a full clinical examination. Students were asked not to tip the sheep they examined in the triangular pens. A ‘teaching session’ was defined as the time taken by a student to examine a sheep. If another student joined during the examination, the time of joining was recorded as the start of a new teaching session and the end of the existing session. If a student returned to examine the same sheep again (even if another student had examined that sheep in the meantime), this was recorded as a new teaching session and the end of the previous session. It was also recorded that this was the second time the student had used the same sheep. If there was more than one person in the triangular pen, this was recorded.

A total of 129 second‐year veterinary undergraduate students participated in the clinical examination classes. Previous training in animal handling had taken place during a practical sheep handling class in their first year. Additionally, some students may have also spent time on a lambing placement as part of their extramural studies (EMS). Before the classes, students were expected to prepare themselves by watching video material demonstrating the clinical examination of sheep. A clinician was available during the classes to assist, although no demonstration was given at the start of each class. At the beginning of the class, students were asked for written consent to be filmed while performing the clinical examination.

### Camera system

Two cameras (Sony Starvis [2MP] model, KH‐778 VR) were used to capture video footage of the ewes. The cameras were placed in the pens before the ewes arrived at the on‐campus teaching hospital. Each camera was attached to a 2.1 m‐tall wooden pole, which was attached to the pillar of the building with cable ties. The cameras were positioned so that the small triangular pens and most of the housing pens could be filmed (Figure 2). The footage was stored on a digital video recorder (Guardian AHD‐DVR‐8G recording system) and downloaded to an external hard drive each day. The data were then backed up to a second hard drive and stored on the university server.

### Behavioural observations

On the day of the clinical examination classes, the maintenance and stress behaviour of the ewes was observed for 1 hour during each of three periods: before teaching, during teaching and after teaching (Table 1), so that the ewes served as their own controls in the period before and after teaching.

**TABLE 1:** Description of approaches used to assess behaviour in study sheep

Time period	Behavioural assessment technique used	Duration of assessment (minutes)	Pen type
Before teaching	Scanning and counts	60	Housing pen
During teaching	Scanning and counts	65–70^a^	Triangle pen
After teaching	Scanning and counts	60	Housing pen

^a^ Actual time used for teaching varied depending on the number of students who examined each sheep and the time the examination took.

To describe the behaviour of the ewes, an ethogram (Table 2) was created by the authors using a combination of published literature on sheep behaviour^23^, ^24^, ^25^, ^26^ and observation of two sheep for 1 hour each during the teaching period. To determine the appropriate time interval for scanning, the same time period and the same two sheep were used. Scanning intervals of 5, 10 and 20 seconds were then used to establish the interval that provided sufficient detail without compromising the quality of the behavioural observations. A scanning interval of 10 seconds was selected to be used as the interval for the maintenance behaviour. This scanning interval was based on the observations made within the teaching period when the animal was being examined by students and was subsequently applied to all three of the periods, even though a less detailed scanning interval could have been used in the period before and after teaching. For the observations of stress behaviour, a scanning interval of 10 seconds was considered too long, as stress behaviour is short‐lived and sporadic, and therefore, continuous observation (i.e., counting stress behaviours) was used to quantify the stress behaviour during all three periods

**TABLE 2:** Ethogram of maintenance and stress and behaviour

Behaviour	Description
*Maintenance behaviour*	
Lying	Sternal position and ventral sternum touches the ground The legs are under or beside the animal
Eating	Eats hay or concentrate feed
Standing	Standing still, and no other behaviours are observed All four feet touch the ground
Ruminating	Rumination (movement of the jaws) while in a standing or lying position
Walking	Moving more than two steps in the forward direction
(Assumed) Urinating	Bending hindlimbs so that the back is pointing towards the ground
Social behaviour	Physical contact with other sheep Includes behaviour such as sniffing or attacking (head butting) other sheep, or resting the chin on other sheep
Other behaviour	Other behaviour that is not described in this ethogram
Not visible	Not visible on video recording
*Stress behaviour*	
Dropping down	Falls to the ground with two legs, only standing on back legs, or completely falls to the ground with four legs (sternal position) while being touched by student/teacher
Escaping	Movement away when touched by a person or because of proximity to a person
Immobilisation	Being frozen/not moving when being touched by students/teacher or when students/teacher are around in the pen No movement of the head visible, or the sheep in a docile posture
Other behaviour	Ewe shows other behaviour that is not described in this ethogram

### Behavioural software

The open‐source programme BORIS, as described by Friard and Gamba,^27^ was used together with Microsoft Excel (Microsoft Corporation, 2022) to record behaviour.

### Statistical analysis

Descriptive statistics and statistical analyses were performed with R Statistical Software version 4.3.3,^28^ using the packages ‘glmTMB’,^29^ ‘DHARMa’,^30^ ‘ggeffects’^31^ and ‘sjPlot’.^32^

For the statistical model of the stress behaviour counts, all the observed stress behaviours (dropping and escaping) were combined to give a total stress behaviour count value. As the stress behaviour was recorded as counts, the distribution was right‐skewed and therefore the Poisson statistical model or the negative binomial linear model was considered the most appropriate choice.^33^

Before the final model was created, univariate analyses were conducted on the following variables: day (1, 2, 3, 4, 5), teaching session within the day (1, 2, 3, 4, ≥5), number of people in the pen (1, >1), housing pen (1, 2), hour of use in clinical examination classes (1, 2).

For the final model, the negative binomial mixed linear model had a lower Akaike information criterion and was therefore selected. This model investigated the influence of the following explanatory variables on the total stress behaviour count (*u*_i_): day (1, 2, 3, 4, 5) (*β*_j_), teaching session within the day (1, 2, 3, 4, ≥5) (*β*_k_), number of people in the pen (1, >1) (*β*_l_), sheep (*β*_m_), the effect of day × teaching session (*ν*_jk_), and the effect of the logarithm of the session duration (minutes) (*γ*_t_). Day 1, sheep 4 and session 1 were used as reference values:

$$\log\;\mu_{\mathrm{ijklmt}}=u_{i}+\beta_{j}+\beta_{k}+\beta_{l}+\left(\beta_{m}\right)+\nu_{\mathrm{jk}}+\gamma_{t}$$

Behavioural scanning analysis data were converted into proportions of the total observations during the time the animal was visible. As the only maintenance behaviour observed during the teaching period was standing, it was decided to exclude the teaching period from the statistical analysis. Beta regression^34^ within a generalised mixed model was used to investigate the differences between time periods (before or after teaching) on the same day. Within the beta regression, the effect of day (1, 2, 3, 4, 5) (*β*_j_), sheep (*β*_k_) and the effect of day × time period were included (*ν*_jk_) together with the proportion of time (*u*_i_). Day 1, sheep 1 and the period before teaching were used as reference values:

$$\mathrm{logit}\;\mu_{\mathrm{ijkl}}=u_{i}+\beta_{j}+\beta_{k}+\nu_{\mathrm{jl}}$$

For lying behaviour, the effect of sheep (*β*_k_) was excluded from the final model as the *p*‐value was above 0.20.

## RESULTS

### Descriptive results of sheep behaviour observations before, during and after teaching

Behavioural observations of the before‐ and after‐teaching periods were conducted 1 hour before and 1 hour after teaching each day, resulting in a total observation of 5 hours before teaching and 5 hours after teaching per sheep (Table 3). During teaching, the total time during which stress behaviour observations could be made was variable, ranging from 18 to 60 minutes. The proportion of time that sheep were out of the detection range of the cameras was 0% before teaching, less than 3% during teaching and more than 22% (for some sheep) after teaching.

**TABLE 3:** Number of recorded minutes per sheep before, during and after teaching

	Sheep 1	Sheep 2	Sheep 3	Sheep 4	Sheep 5	Sheep 6
Before teaching						
Recorded hours	300	300	300	300	300	300
Time animal not visible on camera (%)	0	0	0	0	0	0
During teaching						
Time animals used in classes (min)	243	236	181	176	225	228
Time animal not visible on camera (%)	1.3	2.8	1.3	1.3	1.4	0
After teaching						
Recorded hours	300	300	300	300	300	300
Time animal not visible on camera (%)	11.6	22.9	4.8	1.7	3.4	2.6

The total number of teaching sessions per sheep per day and in total over the 5 days was variable (Table 4); this was partly because students sometimes chose to examine an extra sheep or join another student to examine a sheep together. Stress behaviour was only observed during the teaching period when the sheep were being examined by students or clinicians, and not in the period before or after the teaching period. Maintenance behaviour, other than standing behaviour, was not observed during the teaching period.

**TABLE 4:** Number of teaching sessions per day per sheep

	Day 1	Day 2	Day 3	Day 4	Day 5	Total
Sheep 1	6	5	3	8	7	29
Sheep 2	5	5	3	7	7	27
Sheep 3	5	2	1	5	2	15
Sheep 4	9	0	9	7	5	30
Sheep 5	4	5	5	3	4	21
Sheep 6	2	6	6	4	3	21

### Stress counts

**FIGURE 3:**
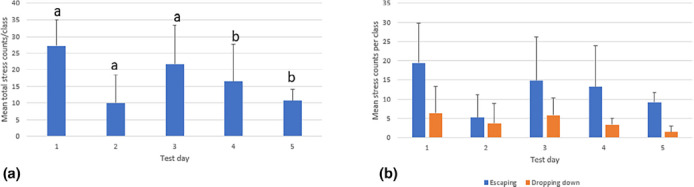
(a) Mean number of stress behaviours observed per class for all sheep per test day. The error bars are standard deviations. The letters indicate a: no significant difference compared to Day 1, b: a significant difference compared to Day 1 (*p* < 0.05). (b) Mean number of stress behaviours observed for all sheep per test day, divided in escaping behaviour and dropping down behaviour. The error bars are standard deviations

Stress behaviour was observed in all teaching classes across all days, with the highest number of stress behaviours (counts per class) observed on the first day (Figure 3). There were no differences in stress behaviour between sheep being used in the first or second hour of the clinical examination classes (*p* = 0.99), nor were there differences in total stress behaviour counts between the two housing pens (*p* = 0.24). Compared to the first day, the total numbers of stress behaviours observed were lower on Day 4 and Day 5 (*p* < 0.05, Figure 3).

**FIGURE 4:**
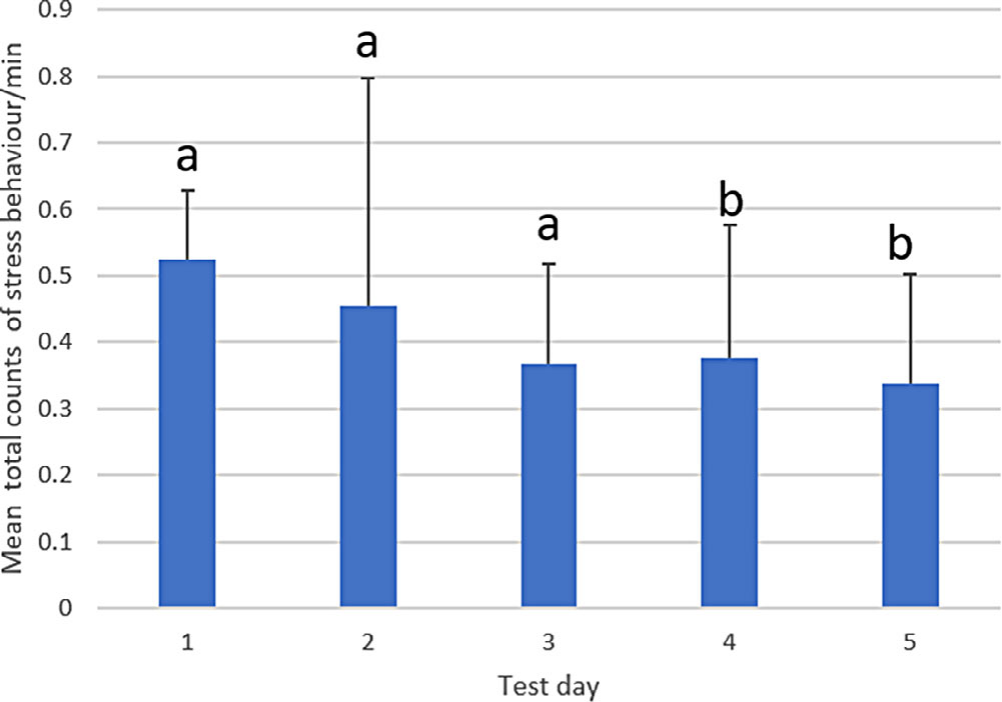
Mean number of stress behaviours observed per minute for all sheep per test day. The error bars are standard deviations. a: no significant difference compared to Day 1, b: a significant difference compared to Day 1 (*p* < 0.05)

**FIGURE 5:**
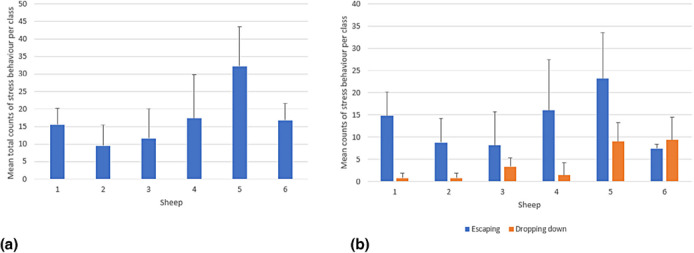
(a) Mean number of stress behaviours observed per class for each sheep over the total teaching period. (b) Mean number of stress behaviours observed divided in escaping behaviour and dropping down behaviour. The error bars are standard deviations

As the animals were not used for the same amount of time on all days, the stress behaviour counts were averaged per minute across all sheep (Figure 4). Overall, there was an effect of session (when a student was performing an examination), where fewer stress behaviours were observed in session 4 (combined across all days) than in other sessions (*p* < 0.05), but the interaction between day and session did not reveal a clear pattern, with considerable variability between day and individual students (Table 5). When there was more than one person in the pen, there was a tendency for fewer stress behaviour counts to be observed than when there was only one person in the pen (*p* = 0.053). Differences in mean total counts of stress behaviour were observed between individual sheep (Figure 5). Sheep 2 showed the least stress behaviour, and sheep 5 showed the most. Escaping was the most frequently observed stress behaviour in all sheep apart from sheep 6, which spent a higher proportion of time dropping down (Figure 5).

**TABLE 5:** Parameter estimates of the effects of number of people, day, session and interaction of day × session on stress counts in negative binomial mixed linear model

	Counts		
Predictors	Estimate	Std. error	*p*‐value
Number of people = 1	Ref		
Number of people >1	−0.72	0.37	0.053
Day 1	Ref		
Day 2	0.043	0.38	0.91
Day 3	−0.58	0.35	0.10
Day 4	−1.31	0.40	**<0.05**
Day 5	−1.10	0.39	**<0.05**
Session 1	Ref		
Session 2	−0.58	0.38	0.13
Session 3	−0.54	0.38	0.15
Session 4	−1.23	0.41	**<0.05**
Session ≥5	−0.54	0.38	0.16
Sheep 5	Ref		
Sheep 1	−0.72	0.21	**<0.05**
Sheep 2	−1.28	0.24	**<0.05**
Sheep 3	−1.01	0.30	**<0.05**
Sheep 4	−0.13	0.22	0.56
Sheep 6	−0.64	0.22	**<0.05**
Day 1 × Session 1	Ref		
Day 2 × Session 1	Ref		
Day 3 × Session 1	Ref		
Day 4 × Session 1	Ref		
Day 5 × Session 1	Ref		
Day 1 × Session 2	Ref		
Day 2 × Session 2	−0.96	0.79	0.22
Day 3 × Session 2	0.24	0.54	0.64
Day 4 × Session 2	1.63	0.60	**<0.05**
Day 5 × Session 2	0.50	0.57	0.38
Day 1 × Session 3	Ref		
Day 2 × Session 3	−0.51	0.59	0.39
Day 3 × Session 3	−0.19	0.57	0.74
Day 4 × Session 3	0.96	0.55	0.083
Day 5 × Session 3	−0.26	0.70	0.71
Day 1 × Session 4	Ref		
Day 2 × Session 4	0.65	0.66	0.32
Day 3 × Session 4	1.27	0.59	**<0.05**
Day 4 × Session 4	1.26	0.65	0.051
Day 5 × Session 4	1.40	0.70	**<0.05**
Day 1 × Session ≥5	Ref		
Day 2 × Session ≥5	0.42	0.66	0.52
Day 3 × Session ≥5	−0.95	0.61	0.12
Day 4 × Session ≥5	0.85	0.57	0.13
Day 5 × Session ≥5	0.54	0.66	0.42

*Note*: Bold font indicates statistical significance (*p* < 0.05).

### Maintenance behaviour

**FIGURE 6:**
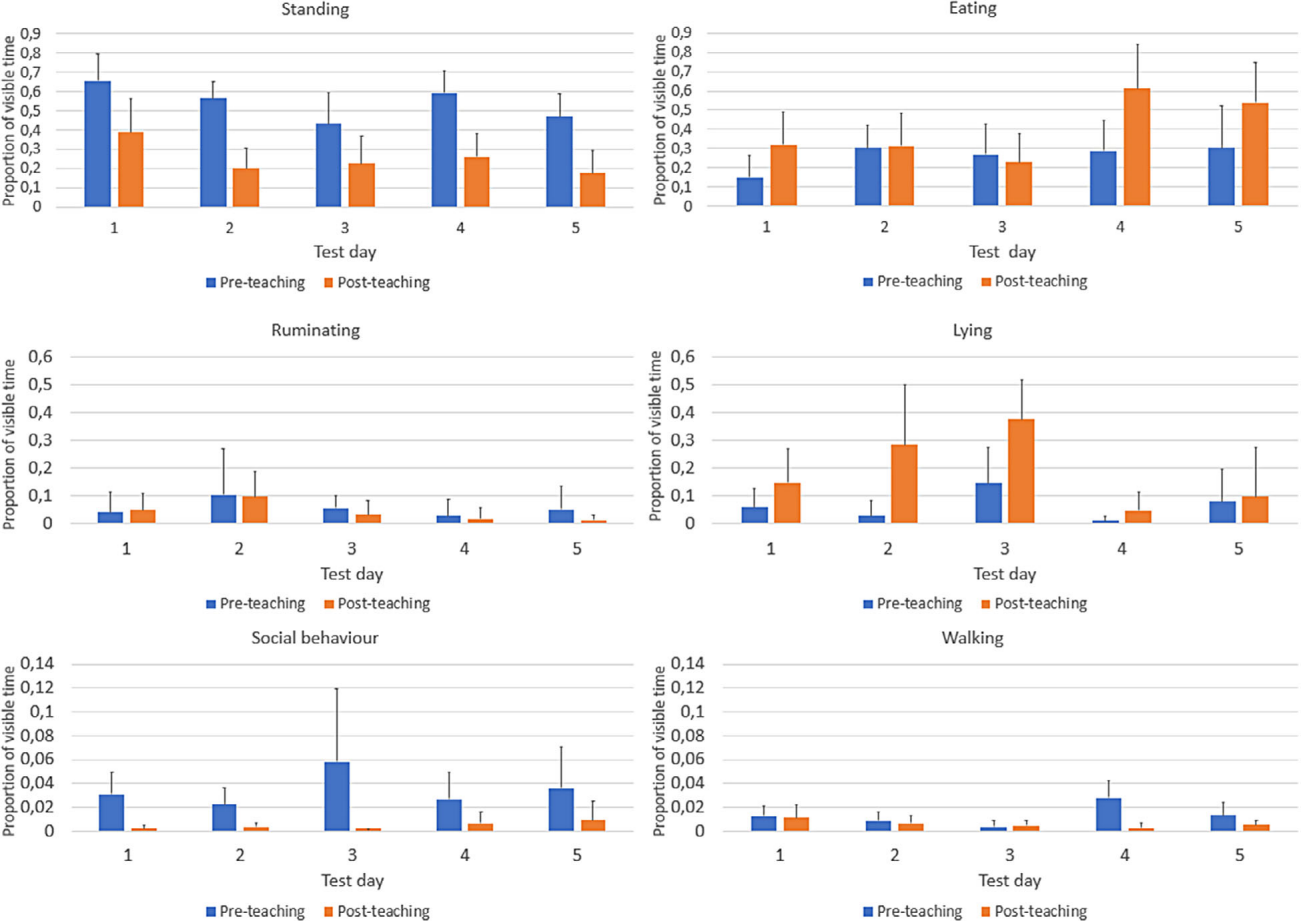
Maintenance behaviour of sheep before and after teaching on each day of the teaching period. The error bars are standard deviations

As the only maintenance behaviour observed during the teaching period was standing, it was decided to exclude the teaching period from this part of the analysis. Therefore, maintenance behaviour was only compared between the periods before and after teaching (as shown in Figure 6). As social behaviour and walking were observed in less than 10% of the time the sheep were visible, these behaviours were only analysed descriptively. An overall difference between before and after teaching was observed for lying, with more time spent lying after teaching on Days 2 and 3 (*p* < 0.05) (Table 6). Differences in eating behaviour between the two periods were observed on Days 4 and 5, when sheep ate more after teaching than in the period before teaching (*p* < 0.05) (Table 7). Overall, standing was observed less on Days 3 and 5 compared to Day 1 (*p* < 0.05), and in all periods after teaching on all days (*p* < 0.05) (Table 8). There was no difference in rumination observed between days or between periods (Table 9). Individual maintenance behaviour differences between sheep were mainly observed for eating and standing behaviour. No individual differences were observed for lying behaviour.

**TABLE 6:** Parameter estimates for the proportion of time sheep exhibited lying behaviour

Predictors	Estimate	Std. error	*p*‐value
Day 1	Ref		
Day 2	−0.44	0.59	0.45
Day 3	0.28	0.59	0.64
Day 4	−0.52	0.59	0.38
Day 5	−0.35	0.59	0.56
Day 1 × Before teaching	Ref		
Day 1 × After teaching	0.61	0.59	0.30
Day 2 × Before teaching	Ref		
Day 2 × After teaching	1.50	0.59	**<0.05**
Day 3 × Before teaching	Ref		
Day 3 × After teaching	1.80	0.58	**<0.05**
Day 4 × Before teaching	Ref		
Day 4 × After teaching	0.29	0.59	0.61
Day 5 × Before teaching	Ref		
Day 5 × After teaching	0.38	0.59	0.52

*Note*: Bold font indicates statistical significance (*p* < 0.05).

**TABLE 7:** Parameter estimates for the proportion of time sheep exhibited eating behaviour

Predictors	Estimate	Std. error	*p*‐value
Day 1	Ref		
Day 2	0.75	0.51	0.13
Day 3	0.58	0.49	0.23
Day 4	0.60	0.49	0.22
Day 5	0.57	0.49	0.25
Sheep 1	Ref		
Sheep 2	−0.63	0.37	0.090
Sheep 3	−1.42	0.39	**<0.05**
Sheep 4	−0.51	0.36	0.16
Sheep 5	−1.35	0.40	**<0.05**
Sheep 6	−1.18	0.38	**<0.05**
Day 1 × Before teaching	Ref		
Day 1 × After teaching	0.87	0.49	0.073
Day 2 × Before teaching	Ref		
Day 2 × After teaching	−0.66	0.51	0.20
Day 3 × Before teaching	Ref		
Day 3 × After teaching	−1.23	0.55	**<0.05**
Day 4 × Before teaching	Ref		
Day 4 × After teaching	1.38	0.47	**<0.05**
Day 5 × Before teaching	Ref		
Day 5 × After teaching	1.15	0.46	**<0.05**

*Note*: Bold font indicates statistical significance (*p* < 0.05).

**TABLE 8:** Parameter estimates for the proportion of time sheep exhibited standing behaviour

Predictors	Estimate	Std. error	*p*‐value
Day 1	Ref		
Day 2	−0.037	0.24	0.13
Day 3	−0.94	0.23	**<0.05**
Day 4	−0.24	0.24	0.31
Day 5	−0.77	0.24	**<0.05**
Sheep 1	Ref		
Sheep 2	0.19	0.20	0.34
Sheep 3	0.90	0.20	**<0.05**
Sheep 4	0.69	0.20	**<0.05**
Sheep 5	0.66	0.20	**<0.05**
Sheep 6	1.43	0.21	**<0.05**
Day 1 × Before teaching	Ref		
Day 1 × After teaching	−1.13	0.23	**<0.05**
Day 2 × Before teaching	Ref		
Day 2 × After teaching	−1.72	0.26	**<0.05**
Day 3 × Before teaching	Ref		
Day 3 × After teaching	−0.97	0.24	**<0.05**
Day 4 × Before teaching	Ref		
Day 4 × After teaching	−1.50	0.25	**<0.05**
Day 5 × Before teaching	Ref		
Day 5 × After teaching	−1.50	0.26	**<0.05**

*Note*: Bold font indicates statistical significance (*p* < 0.05).

**TABLE 9:** Parameter estimates for the proportion of time sheep exhibited rumination behaviour

Predictors	Estimates	Std. error	*p*‐value
Day 1	Ref		
Day 2	−0.084	0.55	0.87
Day 3	0.072	0.57	0.89
Day 4	−0.35	0.56	0.53
Day 5	−0.24	0.59	0.68
Sheep 1	Ref		
Sheep 2	−0.13	0.47	0.78
Sheep 3	−0.13	0.47	0.77
Sheep 4	0.18	0.47	0.70
Sheep 5	1.36	0.45	**<0.05**
Sheep 6	0.42	0.47	0.38
Day 1 × Before teaching	Ref		
Day 1 × After teaching	0.17	0.55	0.74
Day 2 × Before teaching	Ref		
Day 2 × After teaching	0.58	0.58	0.31
Day 3 × Before teaching	Ref		
Day 3 × After teaching	−0.77	0.61	0.20
Day 4 × Before teaching	Ref		
Day 4 × After teaching	−0.15	0.57	0.78
Day 5 × Before teaching	Ref		
Day 5 × After teaching	−0.12	0.59	0.83

*Note*: Bold font indicates statistical significance (*p* < 0.05).

## DISCUSSION

To the authors’ knowledge, this is the first study to investigate the impact of clinical teaching classes on sheep behaviour. This study showed that naïve sheep exhibit stress behaviours with individual variations during clinical examination classes for veterinary students. Fewer stress behaviours were observed on Days 4 and 5, suggesting that habituation to clinical examinations may have occurred. There was an observed change in the demonstration of normal behaviours in the period after teaching, with a greater proportion of time spent either eating or lying down and less time spent standing on some days. These findings could suggest that being used for teaching is stressful and tiring, but it is reassuring that the results suggest that habituation to teaching may be occurring in this context.

The differences in observed stress behaviour counts between the teaching sessions on the same day and between the days could be an indication of the effect of individual differences among students, including variation in experience and approach towards the animals in a clinical examination class, that are likely to affect the behavioural responses of the sheep.^18^ The fact that the students knew that they were being filmed could also have changed their approach to the sheep, although this factor was present on all 5 days. The negative correlation between the number of students in the pen and the number of stress behaviour counts observed could be related to the type of stress behaviour observed, with reduced space available for active stress behaviour when there is more than one student in the pen. In other words, the sheep may still have been stressed but did not have the space to exhibit this behaviour properly.^35^ The influence of limited space on the behavioural expression of sheep was observed by Bøe et al. In this study, the influence of reduced pen size on the lying time of 24 adult ewes was observed, and it was found that reduced pen size resulted in reduced lying time.^36^ Although escape behaviour was not investigated in the present study, the reduced space available due to more than one person being in the pen may have influenced the sheep's decision to attempt to escape.

Together with differences observed between the before and after teaching maintenance behaviours, differences were also observed between the individual sheep in terms of maintenance behaviours. Interestingly, all sheep exhibited increased lying and eating behaviour in the after‐teaching period, which may indicate that the teaching classes are tiring for the sheep and that rest is required afterwards to recover and restore energy. The absence of other maintenance behaviours apart from standing during the teaching period could be another indicator of stress due to teaching.^15^

Given that this teaching event was found to be stressful for the sheep, alternatives to the use of live animals in veterinary education training may be indicated. Various models and simulators have been widely reported in the literature,^37^ but they often lack the high fidelity required to teach a complex skill such as clinical examination. Therefore, live animals are required to teach Day 1 competencies. Using the utilitarian perspective,^38^ the stress induced in these animals may be justified by the prevention of unnecessary stress in other animals, which could be used to justify their use in this context. However, stress should be reduced as much as possible. Training the sheep to become habituated to the clinical examination is an option to help reduce stress during teaching. Several studies have shown that habituation of sheep to a particular stressor is possible. For example, Raoult et al found that habituation to a positive stimulus occurred in sheep, although there was less of an habituation effect if the stimulus was a negative.^39^ Meanwhile, Fonseca et al. found that habituation to people is partially possible; compared to untrained animals, the flight distance to moving people can be reduced if sheep are trained, but the distance to a non‐moving person remained the same.^40^ In contrast, habituation to a stressor was not observed in a study by Atkison et al., in which they used extensively reared 2–3‐month‐old Merino lambs to test whether their fear in general and fear of people would decrease over time. In their study, in which several tests were performed several days a week for seven consecutive weeks, no reduction in fear of people was observed.^41^ One possible reason for the difference between the findings of the present study and the study by Atkinson et al. may be that the animals are touched by people in the clinical examination classes reported in the current study, whereas in Atkinson et al.'s tests, the lambs were only approached. Another possible explanation for the difference is that Atkison et al.'s study used lambs, whereas the current study used adult ewes. Alternatively, instead of habituation, the sheep in the present study could have learned helplessness and therefore shown less stress behaviour. Although this may have been possible, a decrease in maintenance behaviour, especially food intake, would have been expected after the teaching period,^42^ which was not the case in the current study.

As more veterinary schools train larger numbers of veterinary students, we need to ensure the highest standards of care for the animals we use while also providing the best possible training for students. Although this study used only six sheep, had different students examining the sheep each week and did not determine how long a recovery period needed to be, this study could still be used to inform veterinary educators. Specifically, stress associated with use in teaching could be reduced by selecting sheep that show less stress behaviour and by building in a period of habituation for sheep before they are fully used in teaching classes. In addition, as teaching classes could be tiring for sheep, they should be given sufficient time to recover after the class. However, further work is needed to determine the time required for sheep to fully rest and recover following a teaching intervention.

## AUTHOR CONTRIBUTIONS

All authors contributed to the conceptualisation of the study. Sander Prins collected the data. Sander Prins, Jayne Orr, Kathryn Ellis and Dorothy McKeegan analysed the data. Sander Prins drafted the original manuscript, supervised by Jayne Orr, Kathryn Ellis and Dorothy McKeegan. Jayne Orr, Kathryn Ellis and Dorothy McKeegan had input into revising the manuscript. All authors read and approved the final manuscript.

## CONFLICT OF INTEREST STATEMENT

The authors declare they have no conflicts of interest.

## FUNDING INFORMATION

The authors received no specific funding for this work.

## ETHICS STATEMENT

The methods outlined in this study received ethical approval from the Animal Welfare and Ethics Committee of the School of Veterinary Medicine at the University of Glasgow (EA 52/21).

## Data Availability

The data that support the findings of this study are available from the corresponding author upon reasonable request.
